# Early life factors and oral microbial signatures define the risk of caries in a Swedish cohort of preschool children

**DOI:** 10.1038/s41598-024-59126-z

**Published:** 2024-04-11

**Authors:** Carsten Eriksen, Katarina Boustedt, Si Brask Sonne, Jovanna Dahlgren, Karsten Kristiansen, Svante Twetman, Susanne Brix, Josefine Roswall

**Affiliations:** 1 Department of Biotechnology and Biomedicine, Technical University of Denmark, Kgs. Lyngby, Denmark; 2 Department of Paediatrics, Sahlgrenska Academy, University of Gothenburg, Gothenburg, Sweden; 3 Maxillofacial Unit, Halland Hospital, Halmstad, Sweden; 4 Laboratory of Genomics and Molecular Biomedicine, Department of Biology, University of Copenhagen, Copenhagen, Denmark; 5 Department of Pediatrics, Queen Silvia Children’s Hospital, Västra Götalandsregionen, Gothenburg, Sweden; 6 BGI-Shenzhen, Shenzhen, 518083 China; 7 Qingdao-Europe Advanced Institute for Life Sciences, Qingdao, 266555 Shandong China; 8 Department of Odontology, Faculty of Health and Medical Sciences, University of Copenhagen, Copenhagen, Denmark; 9 Department of Paediatrics, Halland Hospital, Halmstad, Sweden

**Keywords:** Ecology, Microbiology, Biomarkers, Diseases, Health care, Medical research, Risk factors

## Abstract

The oral cavity harbors complex communities comprising bacteria, archaea, fungi, protozoa, and viruses. The oral microbiota is establish at birth and develops further during childhood, with early life factors such as birth mode, feeding practices, and oral hygiene, reported to influence this development and the susceptibility to caries. We here analyzed the oral bacterial composition in saliva of 260 Swedish children at two, three and five years of age using 16S rRNA gene profiling to examine its relation to environmental factors and caries development at five years of age. We were able to assign the salivary bacterial community in each child at each time point to one of seven distinct clusters. We observed an individual dynamic in the development of the oral microbiota related to early life factors, such as being first born, born by C-section, maternal perinatal antibiotics use, with a distinct transition between three and five years of age. Different bacterial signatures depending on age were related to increased caries risk, while *Peptococcus* consistently linked to reduced risk of caries development.

## Introduction

The oral bacterial communities consist of a diverse range of bacteria comprising more than 700 unique species with a composition reflecting the different ecological niches found in the oral cavity^1^. The acquisition and establishment of the oral bacterial communities have been addressed in several prospective investigations in recent years^2–5^. Thus, it is clear that the compositional pattern is heterogenic, but shifts as the child matures, starting from birth with early colonizers (*Streptococcus* and *Veillonella*) while certain Gram-negative genera, such as *Neisseria,* appear after one year of age^6,7^. In the very early stages, perinatal factors and circumstances such as mode of delivery, breastfeeding, and prescribed antibiotics are determinants for the overall distribution of taxa and relative abundance of specific species^2,4,8^. Once established, the composition of the oral bacterial communities remains relatively stable over time^9^. However, ecological perturbations that affect the structure and function can induce dysbiosis in the local environment and lead to outbreak of oral diseases such as dental caries^10^.

Early childhood caries is a biofilm-mediated, sugar-driven, multifactorial, non-communicable disease detected before six years of age^11^. Cross-sectional studies have demonstrated an enrichment of species with an acid-producing and acid-tolerating phenotype in biofilms overlying caries lesions^12^. For causality however, longitudinal trials provide best evidence since these have the advantage of determining temporal changes in the bacterial community prior to the caries diagnosis. Such data are available from prospective birth cohorts in which children have been sampled repeatedly over the preschool years^6,7,13–15^. A common observation was that dental caries development seems associated with diverging microbial composition over time, with distinct differences in the composition of the oral bacterial communities between caries-active and caries-free children. Interestingly, the discriminatory role of *S. mutans* and several acid tolerating *Actinomyces/Bifidobacterium* species was a main feature although these species account for only a tiny fraction of the bacterial communities^6,12^. In the dental biofilm, newly described species such as *Scardovia wiggsiae*, *Slackia exigua,* and *Granulicatella elegans* were detected in caries-active children, while commensals like *Streptococcus cristatus, Streptococcus gordonii, Streptococcus sanguinis, Corynebacterium matruchotii* and *Neisseria flavescens* were overabundant at non-carious tooth surfaces^13^. Another study found *Rothia mucilaginosa, Streptococcus* sp., and *Veillonella parvula* to be important biomarkers of risk for caries onset in preschool children^14^.

In longitudinal trials, the salivary bacterial community is of particular interest; firstly, saliva samples can be considered as a representation of various ecological habitats in the oral cavity and, secondly collection of saliva is a non-invasive procedure and thereby convenient for young children. For example, one study showed that the salivary levels of the genera *Atopobium*, *Megasphaera,* and *Veillonella* increased significantly in preschool children before the development of dental caries^15^. Another study found that the saliva bacterial community was diverse already two days after birth and underwent transformations up to five years of age and beyond, with fluctuations possibly reflecting age-related environmental influences^5^. We have previously reported on the influence of perinatal and metabolic risk factors, as well as family and nursing determinants on early childhood caries development in a prospective cohort study^16,17^. In connection with the oral examinations at two, three and five years of age, saliva samples were collected and analyzed. This gave us the opportunity to deepen the insight into the longitudinal maturation of the oral bacterial community during the preschool years and its possible role in caries etiology. The primary aim of the present study was therefore to display ecological changes, including taxa profiles and community structures of the oral salivary bacterial community from two to five years of age. A second aim was to evaluate the microbial diversity in relation to selected environmental exposures such as mode of delivery and infant feeding, and the development of early childhood caries.

## Results

### Oral microbial community structure during early childhood

**Figure 1:**
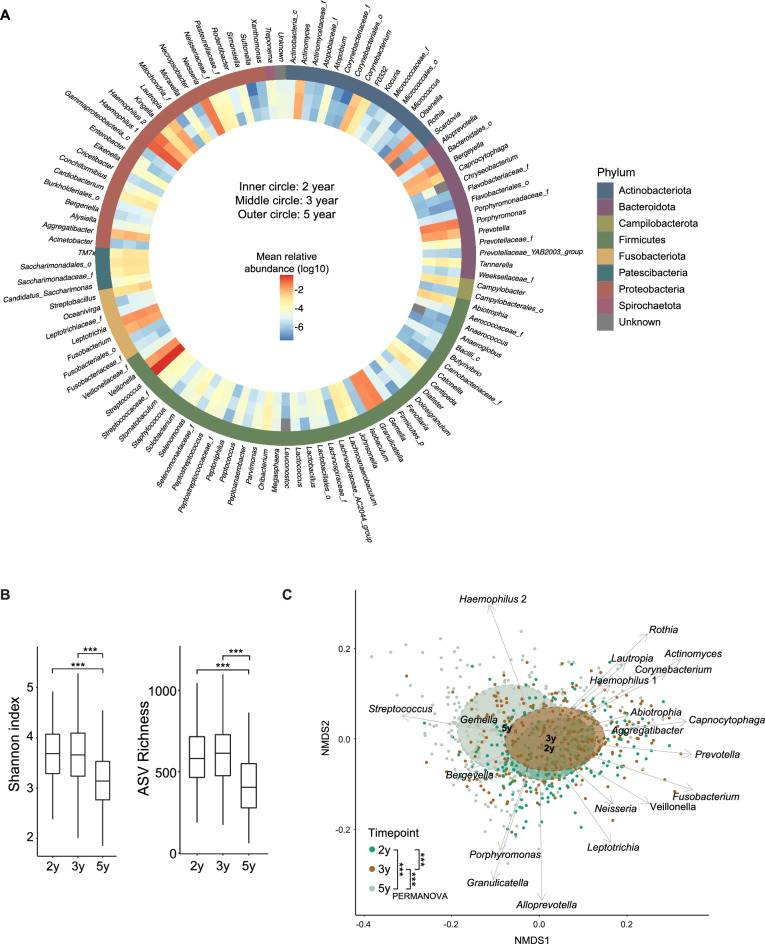
Oral salivary microbial community structure during early childhood. (**A**) The inner, middle, and outer ring represent the mean relative abundance of taxa in the saliva microbiota from children at two, three and five years of age, respectively. (**B**) Shannon index and ASV richness at the three time points are represented by boxplots. The Wilcoxon rank-sum test was used to compare groups with ***, *P* < 0.001. C) A NMDS of Bray–Curtis dissimilarities at all time points. Circles represent the covariance centered on the mean of each time point. PERMANOVA was used to test differences in beta diversity between age groups with ***, *P* < 0.001. Top 20 most abundant taxa across samples are correlated to the first and second dimension and represented by arrows. The length of arrows corresponds to r^2^-values. The bacterial abundance is aggregated to genus level. If genus-level information was missing, the taxa is named after the lowest level available and annotated with “f” or “o" for family- or order-level, respectively. N = 260 for each time point.

The oral bacterial composition in saliva was examined at two, three and five years of age in a longitudinally followed cohort consisting of 260 Swedish children^18^ using 16S ribosomal RNA (rRNA) gene amplicon sequencing of the V3-V4 region. In the 780 samples, we identified a total of 6978 amplicon sequence variants (ASVs), corresponding to 115 unique taxa (Fig. 1A, where the inner, middle, and outer rings depict the mean relative abundance of each of the 115 genera at age two, three and five years, respectively, Supplementary Table 1). Several genera were present and dominated the community structure across the years, with the most abundant being *Streptococcus*, *Haemophilus* (annotated as *Haemophilus* 1 and 2 in the SILVA database with *Haemophilus* 2 being more abundant at 5y compared to 2y and 3y), *Neisseria*, *Gemella* and *Porphyromonas*. The diversity, characterized as the Shannon index and ASV richness, dropped significantly at five years of age, as compared to the two earlier time points (Fig. 1B). At the genus level, we observed a slight, but significant shift in beta-diversity (Bray–Curtis dissimilarity) from two to three years of age (Fig. 1C, PERMANOVA, 2y vs 3y r^2^ = 0.008, *P* = 0.001) with communities characterized by 19 genera. By 5y, compared to 2y and 3y, we observed a less diverse community characterized by four genera *Haemophilus 2*, *Bergeyella*, *Gemella,* and *Streptococcus,* which dominated the oral bacterial community at five years of age (Fig. 1C**,** PERMANOVA, 2y vs 5y, r^2^ = 0.065, *P* = 0.001, 3y vs 5y, r^2^ = 0.055, *P* = 0.001).

### Temporal shifts of the oral salivary bacterial community

**Figure 2:**
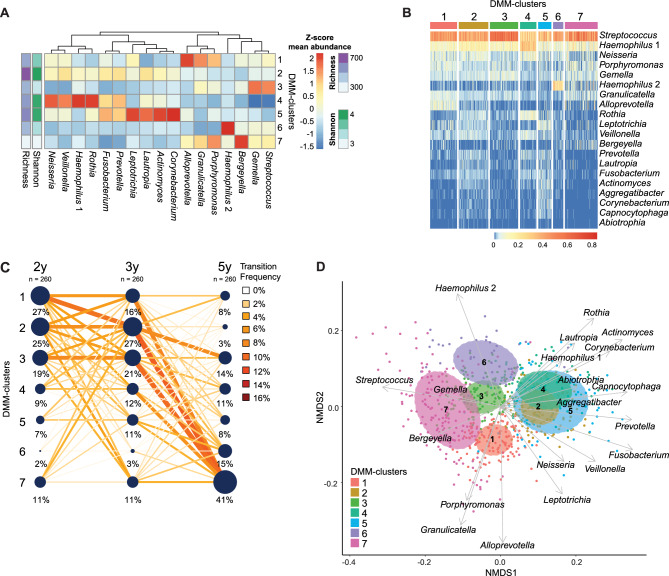
Temporal shifts of the oral salivary microbiota community. DMM was used to cluster the saliva microbiota from children (N = 260) into seven clusters. (**A**) Heatmap showing Z-scored mean relative abundances of the top 10 taxa in each cluster – corresponding to 17 taxa accounting for 96.6% of the mean abundance across children. Mean richness and Shannon index of each cluster are shown to the left of the heatmap. Taxa are ordered by complete hierarchical clustering. (**B**) Heatmap of top 20 most abundant genera in the saliva microbiota of children split according to clustering (**C**) Transition model showing the movement of children between clusters over time. The size of clusters is indicative of the number of children in a cluster, while the width and color of connecting lines indicate the percentage of children following the same trajectory between time points. (**A**, **D**) NMDS of Bray-Curtis dissimilarities at all time points (each N = 260). Circles represent the covariance centered on the mean of each cluster. Top 20 most abundant taxa across samples are correlated to the first and second dimension and represented by arrows. The length of arrows corresponds to r^2^-values.

To investigate temporal shifts of the salivary bacterial community, we made a Dirichlet-Multinomial modelling (DMM) using all 3 × 260 samples as input (Supplementary Fig. 2). This enabled us to assign the salivary bacterial community in each child and time point to one of seven identified clusters. To identify which bacterial taxa that best defined each of these clusters, we identified the top 10 most abundant bacteria in the children of each cluster, which resulted in 17 unique bacterial genera (Fig. 2A). Each of the 17 cluster-defining bacteria is part of the top 20 most abundant bacterial genera found across children at all time points (Fig. 2B), and accounted for an average of 96.6% of the relative abundance in all saliva samples.

After assigning bacteria to each cluster, it became apparent that cluster 1 was best defined by *Alloprevotella*, *Granulicatella*, *Porphyromonas*. Cluster 2 was defined by many genera that were equally distributed in abundance and was the cluster displaying the highest richness and Shannon index amongst the seven clusters. Cluster 3 was the third most dominant cluster at age two and three years, and the one holding the lowest microbial diversity at this age. *Streptococcus* and *Gemella* dominated cluster 3. Cluster 4 and 5 were each dominated by six bacterial genera, with *Prevotella* and *Fusobacterium* overlapping between the two. *Haemophilus* 2 was the sole bacterial taxon to define cluster 6, while cluster 7 was defined by *Bergeyella* and *Porphyromonas*, and four other bacterial genera, including *Streptococcus*.

By illustrating how children transition between clusters over time, we demonstrate that the salivary bacterial community structure within children is subject to marked fluctuations over time (Fig. 2C). Most children exhibited a dominance of bacteria within clusters 1 to 3 at two and three years of age and progressed towards especially cluster 6 (from 2 to 15%) and 7 (from 11 to 41%) at five years of age. *Streptococcus* dominated the salivary bacterial community of all clusters, and the same applied to *Haemophilus* 1, although the latter was most abundant in cluster 4, while *Haemophilus* 2 was prevalent in cluster 6. By the use of non-metric multidimensional scaling (NMDS) of the Bray–Curtis dissimilarities, we illustrated how similar the children of each cluster were to each other, with cluster 1, 3, 6 and 7 clearly separating from other clusters, while cluster 2, 4 and 5 appeared more similar (Fig. 2D).

### Pre- and early post-natal environmental factors affect the salivary microbial community structure

**Figure 3:**
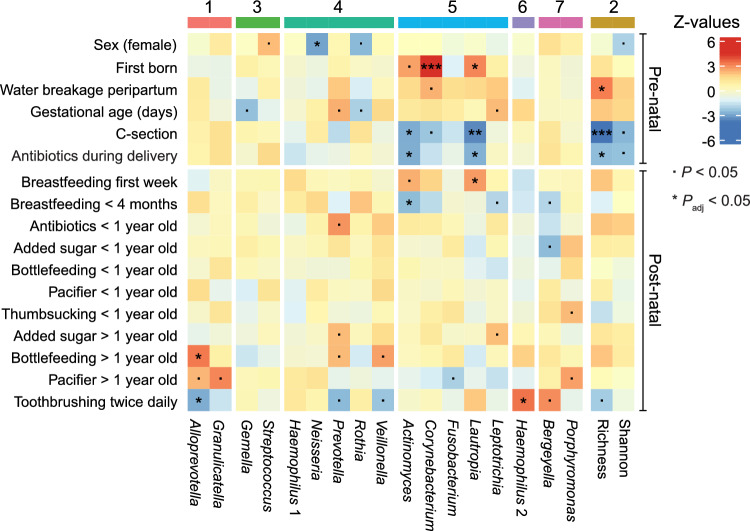
Early environmental factors affect oral salivary microbial community structure. Heatmap representing relations between DMM cluster-defining taxa and sex as well as pre- or post-natal environmental factors in addition to Shannon index and richness (each N = 260). Z-values represent test statistics of coefficients for generalized linear mixed models modelled over a negative binomial distribution or linear mixed models for Shannon index and richness. Z-values show the number of standard deviations the value is from the mean of the distribution. Negative scores indicate that the value lies below the mean and positive values are above the mean. Significant adjusted *P*-values are labeled as following: *: *P* < 0.05, **: *P* < 0.01, ***: *P* < 0.001. *P*-values significant before adjustment are labeled with a dot.

We next aimed to define pre and early postnatal environmental factors associated with each of the seven salivary microbial community clusters. The different environmental factors and their distribution are presented in Table 1. We found that sex influenced the overall bacterial community diversity (PERMANOVA: r^2^ = 0.003, *P* = 0.03), where the oral microbiota in females compared to males exhibited lower diversity and lower abundance of the cluster 5-defining bacterial genus, *Neisseria*. Among prenatal factors, being the first-born child, water breakage prior to delivery, delivery by caesarean section (C-section), and administration of antibiotics to the mother during delivery differentially associated with salivary bacterial abundances, diversity, and richness later in life (Fig. 3). Being first born was associated with increased relative abundance of two cluster 5 bacterial genera, *Corynebacterium* and *Lautropia*, in addition, water breakage prior to delivery associated with increased salivary bacterial community diversity. C-section was linked to lower relative abundance of the cluster 5 dominating bacterial genera, *Actinomyces* and *Lautropia,* and to lower richness of the salivary bacterial community. We found a reduced relative abundance of *Actinomyces* and *Lautropia,* and lower richness in children of mothers who received antibiotics according to the Swedish guidelines (see methods) during delivery, resembling that of C-section children.

**Table 1:** Descriptive data including maternal and infant pre and postnatal factors given as mean (SD) or % (N).

Prenatal factors	N (total)	
Mothers age, years, mean (SD)	260	31.48 (4.78)
Maternal BMI in early pregnancy, mean (SD)	249	24.77 (5.20)
Childs BMI at birth, mean (SD)	255	13.75 (1.53)
Gestational age, days, mean (SD)	260	278.0 (11.62)
Sex, female, % (N)	260	52% (134)
First born, yes, % (N)	256	93% (240)
Water breakage prepartum, yes, % (N)	259	79% (204)
C-section, yes, % (N)	259	34% (87)
Antibiotics during delivery, yes, % (N)	260	21% (56)

Postnatal factors		
Feeding		
Exclusively breastfed first week, yes, % (N)	226	69% (155)
Breastfed less than 4 months, yes, % (N)	257	20% (52)
Any breastfeeding at 1 year of age, yes, % (N)	256	11% (25)
Use of bottle for feeding (i.e. breastmilk, formula or cereal drink)		
Until 1 year of age, yes, % (N)	242	90% (218)
At 2 years of age, yes, % (N)	211	67% (141)
At 3 years of age, yes, % (N)	209	43% (90)
Intake of added sugars		
At 1 year of age, yes, % (N)	260	62% (162)
At 2 years of age, yes, % (N)	260	74% (192)
At 3 years of age, yes, % (N)	260	74% (193)
Antibiotics		
Any antibiotics until 1. year, yes, % (N)	260	23% (59)
Pacifier use		
Less than 1 year of age, yes, % (N)	238	89% (213)
At 2 years of age, yes, % (N)	211	73% (154)
At 3 years of age, yes, % (N)	209	35% (74)
Thumb sucking		
At 1 year of age, yes, % (N)	238	59% (140)
At 2 years of age, yes, % (N)	211	4% (8)
At 3 years of age, yes, % (N)	209	4% (9)
Tooth brushing twice daily		
At 2 years of age, yes, % (N)	260	77% (202)
At 3 years of age, yes, % (N)	259	87% (226)
At 5 years of age, yes, % (N)	259	91% (235)
Tooth brushing by adults		
At 2 years, yes, % (N)	260	87% (227)
At 3 years, yes, % (N)	256	87% (223)
At 5 years, yes, % (N)	257	82% (213)

All prenatal factors and postnatal antibiotics to the child until 1 year of age retrieved from medical records/register and all postnatal factors by parental questionnaire at reported age.

We identified less strong associations between the salivary bacterial community structure and postnatal factors, but breastfeeding habits, bottle-feeding, and tooth brushing frequency were all associated with presence or absence of distinct cluster-defining salivary bacteria. Breastfeeding during the first week of life was associated with higher relative abundance of the cluster 5-defining bacterial genus *Lautropia*, while breastfeeding for less than four months resulted in reduced relative abundance of *Actinomyces*. Bottle-feeding beyond one year of age was associated with increased relative abundance of the cluster 1-defining genus *Alloprevotella*. We also found that tooth brushing twice a day enhanced the relative abundance of the cluster 6-defining bacterial taxon *Haemophilus* 2 but reduced the relative abundance of the cluster 1-defining bacterial genus *Alloprevotella*.

### Environmental factors and oral bacteria of importance for caries onset until five years of age

**Figure 4:**
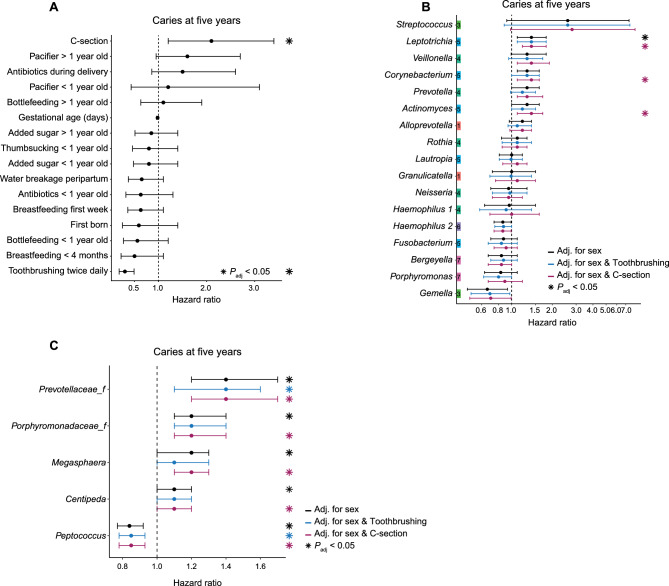
Environmental factors and oral bacteria of importance for caries onset until five years of age. Cox proportional hazard models showing risks associated with environmental factors or specific bacteria and later onset caries at five years of age. (**A**) Hazard ratios and 95% confidence intervals for pre- and post-natal environmental factors in relation to later onset caries at five years of age. All hazard ratios were adjusted for sex. (**B**) Hazard ratios and 95% confidence intervals for cluster-defining bacteria in relation to later onset caries at five years of age. (**C**) Hazard ratios and 95% confidence intervals for oral bacteria in relation to later onset caries at five years of age. Only bacteria significant after adjustment for sex are shown. B + C) Green and purple hazard ratios were adjusted for tooth brushing habits or C-section, respectively, in addition to sex. Significant adjusted *P*-values are labeled by an asterisk. For all analyses, N: 2y = 258, 3y = 249, 5y = 260; total = 767.

We next aimed to examine if any of the environmental early-life factors or the salivary microbial community were associated with an increased or reduced risk of developing caries by the age of five. Caries was expressed as the proportion of children with any tooth with a cavitated or non-cavitated (initial) carious lesion. Among the 260 children, two had developed caries by two years of age, 11 at three years of age, and 52 by the age of five. We generated Cox proportional hazard models for each factor and used caries at five years as the outcome. Tooth brushing twice a day lowered the risk of developing caries until five years of age (HR: 0.3 (0.18–0.49), Fig. 4A). Children born by C-section (N = 87) had an increased risk of developing caries until five years of age (HR: 2.1 (1.2–3.4)), thus, 26 children (30%) born by C-section had developed caries by the age of five. None of the other pre- or postnatal factors were significantly associated to caries development at five years of age.

When focusing on the association between the cluster-defining bacterial taxa and risk of caries development until five years of age, we found that cluster 5-associated *Leptotrichia* associated with an increased the risk of developing caries until five years of age (Fig. 4B). This remained significant after adjusting for tooth brushing frequency and C-section. After adjusting for C-section, two other cluster 5-defining genera, *Corynebacterium* and *Actinomyces*, were also found to associate with an increased risk of caries at five years of age.

As few cluster-defining bacterial genera associated to a risk of caries development until five years of age, we also examined if any other oral bacterial taxa were associated with caries development, independently of the age at which they appeared in the saliva. We identified members of the *Prevotellaceae* family, members of the *Porphyromonadaceae* family, *Megasphera* and *Centipeda* to be associated with an increased the risk of developing caries until five years of age, while *Peptococcus* associated with a reduced risk (Fig. 4C). Adjusting for tooth brushing frequency, we found that the increased caries risk remained significant for members of the *Prevotellaceae* family, while adjustment for C-section did not affect the association between caries and individual bacterial taxon.

### Specific oral bacteria in early life predict caries development at five years of age

**Figure 5:**
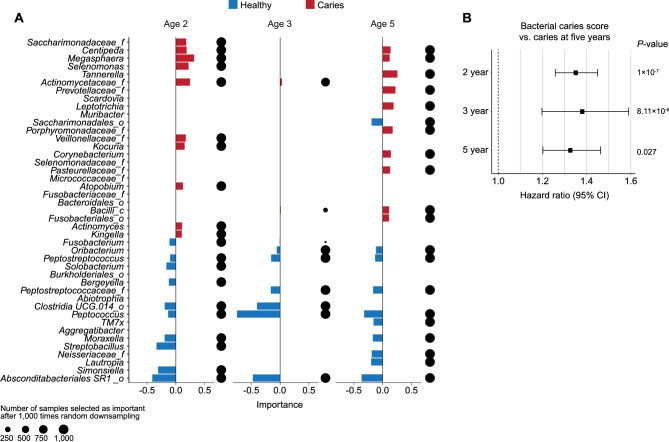
A subset of oral bacteria in early life predicts caries development at five years of age. A sPLS-DA was used to distinguish the salivary bacterial community at two, three and five years between children who did and did not develop caries at five years. Each child was assigned a bacterial caries score. For balanced model prediction, the number of samples from children without caries was randomly downsampled to the number of children who developed caries after that year (2y: N = 50, 3y: N = 41) or had caries at 5y (N = 52). Downsampling was repeated 1,000 times, and results averaged. (**A**) Top 20 most important bacteria at 2, 3 and 5 years of age for discriminating between children who did not and children who developed caries at 5 years of age. The dot size indicates how many times a given bacterium was selected during the 1,000 times repeated downsampling of samples from children without caries. (**B**) Hazard ratios and 95% confidence intervals for bacterial caries scores at two (N = 258), three (N = 249) and five years (N = 260) in relation to later onset caries at five years of age. All hazard ratios are adjusted for sex.

We next investigated if the presence of certain oral bacterial taxa enabled prediction of the risk of caries development in children. To identify the most pro-cariogenic bacterial taxa at each sampling time point, we constructed a cross-validated sparse partial least square discriminant analysis (sPLS-DA) model for each of the three age groups (2y, 3y and 5y) and used caries at five years as the outcome. The sPLS-DA-selected oral bacterial taxa at 2 years of age were found to be slightly better in predicting caries development at five years (AUC = 0.84) than the oral bacterial communities at 3 and 5 years of age (AUC = 0.81 and = 0.78, respectively, Supplementary Fig. 3, Supplementary Table 2). In general, the taxa that were most predictive for caries development changed over time (Fig. 5A), but *Peptococcus* was consistently identified to reduce the risk of caries development. We then calculated a bacterial caries score for each child at each time point based on the models. The individual bacterial caries scores were used to predict the risk of developing caries at five years of age using a Cox proportional hazard model. This showed that a high bacterial caries score at two and three years of age was associated with later onset caries at five years (HR at 2 years: 1.35 (1.26–1.45), HR at three years: 1.38 (1.20–1.59), Fig. 5B). Additionally, the bacterial score at five years of age was also capable of separating children with caries from non-caries at five years of age (HR at five years: 1.32 (1.20–1.46)). Overall, this analysis demonstrated that the structure of a child’s salivary bacterial community in early life predicts the later risk of caries development at five years of age.

### Tooth brushing as a mediator in lowering the bacterial caries score and thereby the risk of caries development

**Figure 6:**
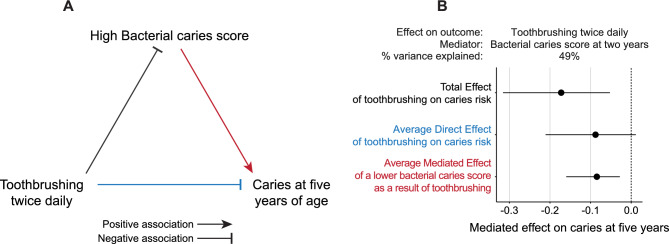
Tooth brushing as a mediator in lowering the bacterial caries score and thereby the risk of caries development. (**A**) Schematic representation of the mediation analysis model structure with tooth brushing frequencies as the predictor, the bacterial caries scores as the mediator, and caries at five years as the outcome. (**B**) Mediation analysis showing that the effect of tooth brushing habits at two years on later caries development at five years is mediated via the effect of tooth brushing on the bacterial caries score (N = 258). All tests were adjusted for sex.

We used a mediation analysis to examine the causal inference of tooth brushing twice daily on the bacterial caries score at two years of age and caries development at five years (Fig. 6A). The analysis revealed that 49% (*P* = 0.006) of the effect of tooth brushing at two years of age on the risk of caries at five years was mediated via lowering of the bacterial caries score (Fig. 6B). This implies that tooth brushing makes the oral bacterial community less cariogenic, and thereby lowers the risk of caries development. At 3 years of age, only 12.6% of the effect of tooth brushing on caries at five years of age was mediated via lowering of the bacterial caries score, and this was not statistically significant.

## Discussion

Here we characterized the bacterial composition in saliva from 260 Swedish children at two, three and five years of age and its relation to peri- and postnatal factors and early childhood caries. According to a recent systematic review, there are only two previous prospective birth cohorts available that address this topic^19^. In this study, participants were enrolled before birth and perinatal and medical data were extracted from hospital records. In addition, self-reported information on family characteristics, behavioral factors, as well as nursing and dietary habits, were captured continuously during the 5-year course to minimize recall bias. We sampled and analyzed unstimulated whole (mixed) saliva as a proxy for the oral bacterial community as a practical non-invasive procedure considering the young age of the children and their ability to cooperate. As saliva does not harbor endogenous bacteria, the salivary bacterial community constitutes a compilation of bacteria shed from all oral surfaces, including the tongue and the throat^4,20^. It is however important to point out that the low-volume saliva samples do not fully mirror biofilm samples collected directly from the teeth, and thereby most relevant for caries development. For example, one recent study found enrichment of cariogenic species in samples from teeth, while fungal species were more abundant in oral swab samples^21^. The spatial structure of the bacterial communities (biogeography) with localized rotund corona-like arrangements may also have direct implications for the initiation of dental caries^22^. Of note, all samples were collected with the same technique throughout the study and could thereby be considered as internally comparable. In addition, it should also be noted the use of a swab placed under the tongue of the infants might constitute a potential limitation, as this protocol may not provide a full representation of the bacterial composition of saliva collected using other protocols.

Our present findings concerning the community structure and temporal shifts were partly in support and partly in conflict with previous findings. The observation that the diversity dropped between 3 and 5 years is in contrast to previous reports^5,6^, while others found no significant changes with age^14^. On the other hand, it has been shown that the oral microbiota of children displays a higher diversity than that of adults^23^. In this context, it is important to underline that the structure of the bacterial community differed markedly within and between children in this study. One may speculate whether the sharp increase in caries prevalence between three and five years may have influenced the observed reduction in the diversity of the saliva microbiota, since dysbiotic biofilms, preceding the disease, are characterized by a reduced community diversity with enrichment of acid tolerant bacteria^12^. To expand the information on this matter, future studies must be designed with more frequent samplings between 3 and 5 years of age, as this age interval represents a knowledge gap today.

Consistent with past research^5^, we showed age-related temporal shifts in the salivary community and transitions between clusters over time, with a domination of *Streptococcus* in all the defined clusters. We also noted that birth-related events, such as mode of delivery, water breakage, and exposure to antibiotics affected bacterial abundance, α-diversity, and richness up to five years of age^4^. This clearly suggests that very early-life events play a role in shaping the oral bacterial community later in life. Among the postnatal factors, breastfeeding habits and oral hygiene habits seemed to influence the bacterial clusters, which is in concert with previous findings^4,23^. Breast milk contains both gut-derived lactic acid bacteria and oligosaccharides that some bacteria, like *Actinomyces*, are capable of utilizing as a carbon source^24,25^. Consequently, we found a reduced relative abundance of *Actinomyces* in children being breastfed for less than four months. Infrequent tooth brushing (less than twice a day) is known to be associated with an increased chance of incidence or increment of caries in the primary dentition^26^. The fact that we found that tooth brushing frequency influenced the composition of the salivary bacterial community is most likely due to the mechanical disruption of the dental biofilm. In this context, it is of note that tooth brushing was associated by a decrease in the relative abundance of the genus *Alloprevotella* and more surprisingly that bottle-feeding beyond one year of age was associated with an increased relative abundance of *Alloprevotella*, given the observation that *Alloprevotella* in a prospective cohort study of Japanese students was shown to be enriched in individuals who developed caries compared to individuals who did not develop caries^27^.

Almost all children brushed their teeth with fluoride toothpaste, and this may have contributed to inhibit biofilm matrix production and hamper the metabolic activity of certain members of the oral biofilm^28^.

Our microbial findings concerning the onset of dental caries lend support to the emerging understanding that classifies dental caries as a non-communicable disease^29^. During the past decades, caries has been regarded as an infectious, transmissible disease^30^, but here we demonstrate that a blend of functional clusters rather than specific pathogens is associated with caries development.

The structure of the oral bacterial community early in life was clearly associated with caries risk and members of the *Prevotellacease*, *Porphymonadacaeae* and *Veillonellaceae* families have previously been linked to caries^14,31^. The potential impact of *Lepthotricia* was interesting in the light of past findings in which oral hygiene discontinuation was associated with a significant increase in relative abundance of this bacterial genus^32^. The negative relation between *Peptococcus* and caries risk is consistent with a previous study reporting that *Peptococcus,* along with *Rothia and Treponema,* were enriched over a 2-year period in preschool children that remained free from caries^15^*.* These Gram-positive bacteria are part of the normal bacterial community of the mouth and may be important members of health-associated functional clusters in oral biofilms. Further investigations seem therefore justified in order to elucidate their potential protective role in the caries process.

In predicting clinical outcomes in prospective cohort studies, it is important to acknowledge the limitations of not knowing how many children will develop caries, which can result in low number of cases. Still, in the present study 52 out of 260 Swedish children had developed carries by the age of five, meaning that carries developed in 20% of the children, enabling rather robust statistically analyses by downsizing the number of children without carries at each time point to balance the number of children with caries at 5 years of age. Ideally, future studies would benefit from enrollment of more children or by changing the sampling strategy to focus on the period between 3 and 5y, which, based on our results, seems to be a critical window for caries development.

In summary, our longitudinal study of the development of the oral bacterial community of saliva during the preschool age shows that different bacterial community signatures at two and three years of age are related to increased risk of caries at five years of age. Our results point towards a potential for early analysis of the oral bacterial community in predicting future caries during early childhood. However, further studies are clearly warranted to substantiate this notion.

## Materials and methods

### Ethical approval

The longitudinal study design was approved by the Regional Ethical Review Board in Lund, Sweden (44/2008, 2010/362, 2012/483) and a written informed consent was obtained from the legal guardians of all the study participants. All research was performed in accordance with relevant guidelines and regulations.

### Study group

The present study group was derived from children participating in a sub-study (N = 551) of the H^2^GS birth cohort in southwest Sweden^18^. Children were invited for dental examinations and saliva samplings. The parents of 346 children (179 boys and 167 girls) accepted and 336 children attended the first visit at two years of age. Follow-up examinations were carried out at three (N = 302) and five years of age (N = 292), respectively. The main reasons for the attrition were relocation or lack of interest or time. Two children rejected the sampling, three children delivered insufficient saliva for analysis and the samples from nine children were discarded due to technical errors. Thus, the findings reported here were based on 260 children (134 girls, 126 boys) with a complete set of medical and dental data together with successfully analyzed saliva samples at all three time points.

### Data collection

Maternal and birth-related factors were collected through a validated questionnaire at the maternity ward as previously described^33^. In addition, medical data, such as prescription of antibiotics, gestational week, and neonatal hospital care, were extracted from the hospital records. Antibiotics administration during labor and C-section in this cohort was performed according to Swedish guidelines, which include mothers with suspected infection or increased risk of infection in vaginal deliveries (i.e., fever during labor or Group B *streptococcus* colonization during late pregnancy). Mothers delivering with C-section received intraoperative antibiotics only if the C-section was classified as acute or if additional risk factors for postoperative infections were present (i.e., severe obesity). We collected key data covering family characteristics, nursing and feeding habits through comprehensive questionnaires at regular intervals during the project^17^. The oral hygiene routines were captured by structured personal interviews and two calibrated and experienced dentists performed a visual-tactile dental examination according to the WHO criteria^34^. The prevalence of dental caries was expressed as the proportion of children with any tooth with a cavitated or non-cavitated (initial) carious lesion^35^.

### Saliva sampling

Resting mixed whole saliva samples (N = 780) were collected at 2, 3 and 5 years of age in connection with the dental examination. The child was seated in an upright position and asked not to swallow. A sterile cotton bud was then inserted under the tongue until soaked and immediately transferred to a sterile Sarstedt tube. The samples were rapidly frozen to -20 °C and transported along with negative controls to the laboratory in Copenhagen for storage at -80 °C until further processing. The amount of saliva collected with this technique was approximately 0.3–0.4 mL.

### DNA extraction and 16S rRNA gene library preparation

DNA was extracted from the saliva samples using the NucleoSpin 96 Soil kit, (MACHEREY–NAGEL, Germany), following the manufacturer´s instructions with a few modifications. Briefly, samples were transferred to NucleoSpin Bead Tubes containing 700 µL Buffer SL1 and 150 µL Enhancer SX and lysed on a horizontal vortexer (Vortex-genie 2, Scientific industries) for 15 min at full speed. The filtering, binding, and washing of the NucleoSpin Soil Binding Plate were performed as described in the centrifuge processing protocol, with centrifugation at 4,700xg, and elution in 50 µL Buffer SE. Negative controls were added to check the cotton swabs, bead tubes, and Sarstedt tubes. The obtained DNA was then refrozen at − 80 °C and transported to BGI Europe, Copenhagen for 16S ribosomal RNA (rRNA) gene amplicon sequencing of the V3-V4 region. The library preparation was performed as follows: DNA samples were analyzed by fluorometry and agarose gel electrophoresis, if possible 30 ng DNA were used as input. Since this sample type has low DNA yield, this was not always possible, but library preparation was attempted for all samples. DNA was amplified using primers 341F (ACTCCTACGGGAGGCAGCAG) and 806R (GGACTACHVGGGTWTCTAAT). All PCR reactions were carried out in 30 µL reactions with 15 µL of Phusion® High-Fidelity PCR Master Mix (New England Biolabs); 0.2 µM of forward and reverse primers. Thermal cycling consisted of initial denaturation at 98℃ for 1 min, followed by 30 cycles of denaturation at 98℃ for 10 s, annealing at 50℃ for 30 s, and elongation at 72℃ for 30 s and finally 72℃ for 5 min.

After PCR, the libraries were purified using AmpureXP beads (AGENCOURT) to remove unspecific products, and the purified libraries were evaluated by using the Agilent 2100 Bioanalyzer system (Agilent DNA 1000 Reagents) and quantified by Quantitative PCR (EvaGreenTM) before pooling. Except for four samples, library preparation was successful. The qualified libraries were sequenced pair end on the MiSeq System, with the sequencing strategy PE300 (PE301 + 8 + 8 + 301) (MiSeq Reagent Kit) and the read depth was > 30 k reads per sample. For information on total reads per sample see supplementary table 3.

### 16S rRNA Gene Data processing

Reads were analyzed and denoised using DADA2^36^. Resulting amplicon sequence variants (ASVs), were mapped to the 99% identity clustered SILVA database v132^37^ using a naïve Bayes classifier trained on the amplified region, as implemented in DADA2. (Supplementary table 3).

Several control samples were collected as part of the study: the cotton swabs and the sample collection tubes were washed with nuclease-free water, which together with empty tubes from the DNA extraction kit underwent the same procedure and sequencing as the saliva samples. The control samples were used to identify contaminating reads in the samples by the use of the R package decontam (v. 1.12)^38^ using default settings. Overall, the control samples did not contain the same bacteria as the biological samples and had fewer sequencing counts (Supplementary Fig. 1A + B).

After correction for contaminating reads, we filtered out low-abundant ASVs with less than 20 read counts across all samples and ASVs found in less than five samples with an abundance under 1%. In total we obtained 6978 ASVs distributed across 115 bacterial taxa. The bacterial abundance was aggregated to genus level. If genus-level information was missing the taxa was named after the lowest level available and annotated with either “f” or “o" for family- or order-level, respectively.

### Statistics and data analysis

All statistical analyses were performed using R (v4.0.0)^39^. Shannon index, which was used as a measure for alpha-diversity and richness as a measure of number of unique taxa was calculated using the vegan package (v. 2.6–2)^40^. Differences in Shannon index and richness between time points were compared using Wilcoxon rank sum test. Visualization of the overall bacterial communities at the three time points or the DMM clusters were done using Bray–Curtis dissimilarities (beta-diversity) made from Hellinger-transformed relative abundances using the decostand function (vegan) and presented by NMDS in seven dimensions identified by iteratively adding dimensions to find a solution with lowest level of stress, which is a measure of remaining variance not captured by the model. The top 20 taxa were added to the NMDS using the function envfit (vegan). The differences in beta-diversity between time points (2y, 3, and 5y) were tested using a permutational multivariate analysis of variance (PERMANOVA with 999 permutations, using Adonis (vegan). The DMM-based temporal transition modelling of the saliva microbiota was performed using the Dirichlet Multinomial package^41^. The optimal number of Dirichlet components (clusters) were chosen by estimating the goodness of fit using Laplace (Supplementary Fig. 2). Samples from all time points were clustered together, which enabled us to identify how individual children move between clusters over time. The graphical representation was done using an R implemented script from a previous study^42^. Environmental factors were fitted to bacterial counts using negative binomial generalized linear mixed models with logged sequencing depths as offset term to compensate for differences in sequencing depth between samples and children IDs as random effect to include information of repeated sampling in the model using the glmmADMB package (v. 0.8.3.3). Shannon index and richness were fitted to environmental factors using linear mixed models with children IDs as random effect. Associations between environmental factors, individual bacteria or bacterial caries scores and caries at five years were quantified in terms of hazard ratios by Cox proportional hazard models adjusted for sex and clustered by children IDs to include information of repeated sampling, using the survival package. The individual bacteria significantly associated with caries at five years were also adjusted for tooth brushing frequencies or C-section in addition to sex. Since the aim was to identify at each age (2y, 3y and 5y) which oral bacteria contributed the most in separating children who developed caries at 5y from those who did not, we utilized sPLS-DA (a supervised clustering method) from the mixOmics package (v. 6.20)^43^. Input data were log10-transformed relative abundances, using random values sampled from a distribution ranging from 1/10 to 1/2 of the lowest non-zero value as pseudocounts^44^. A separate model was trained for each time point with caries at five years as outcome. Children diagnosed with caries at 2y (N = 2) and 3y (N = 11) were excluded from the respective models. Since the number of prospective caries and non-caries cases led to an unbalanced design (caries cases at 5 years = 52, non-caries = 208), we performed a random downsampling of the caries-free children at 2y, 3y and 5y to match the number of children with caries at 5y, subtracting the number of caries cases at the given year (2y: N = 52–2 = 50, 3y: N = 52–11 = 41, 5y: N = 52). To increase robustness and to avoid potential biases of the random downsampling, the process was repeated 1,000 times and the results were averaged across samples. Within each age group, the samples were subjected to a tenfold cross-validation repeated 100 times to avoid overfitting and to select the most discriminating bacteria. The component values from each of the three age-dependent bacterial risk scores were scaled and combined into a bacterial caries score for each age, the latter explaining how much the bacterial community of each sample resembled the bacterial features associated with caries development at 5y. The final models were evaluated using AUC statistics.. Mediation analysis was performed using the mediation package (v. 4.5.0)^45^, using a model structure with tooth brushing frequencies as the predictor, the bacterial caries scores as the mediator, and caries at five years as the outcome. *P* values were deemed significant using 0.05 as significance level; when appropriate, *P* values were adjusted for multiple testing using FDR with an adjusted significance level of 0.05.

### Supplementary Information

Supplementary Figures.

Supplementary Tables.

## Data Availability

Sequencing data are publicly available in European Nucleotide Archive under project: PRJEB60183. Analysis software including quality control, taxonomy, and tools for analysis of ecological communities are publicly available and referenced.
