# Supplementary material for: Early life factors and oral microbial signatures define the risk of caries in a Swedish cohort of preschool children
Source: Sci Rep. 2024 Apr 11;14:8463. doi: 10.1038/s41598-024-59126-z (PMC11009336; doi:10.1038/s41598-024-59126-z)
Supplement: Supplementary file 1 — Supplementary Figures. [file 41598_2024_59126_MOESM1_ESM.pdf]

Supplementary Figure 1

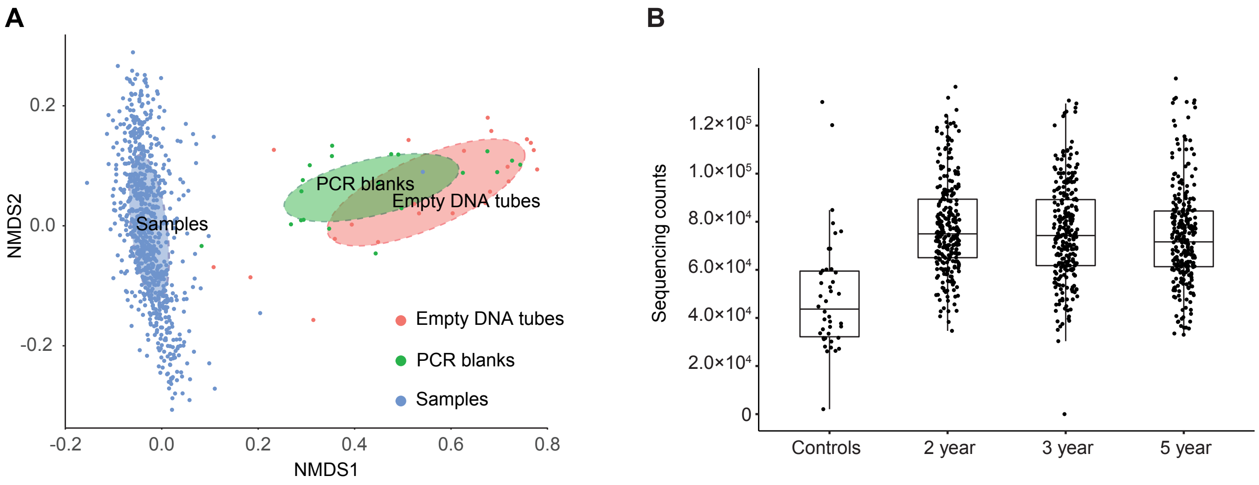

Supplementary Figure 1. Samples and technical controls. A) NMDS performed on the Bray-Curtis distances from children samples and technical laboratory controls in the form of PCR blanks (N = 19) and empty DNA tubes (N = 22) from the DNA purification procedures. B) Boxplot showing the number of sequences identified in children samples at 2y, 3y and 5y compared to those found in the laboratory controls.

Supplementary Figure 2

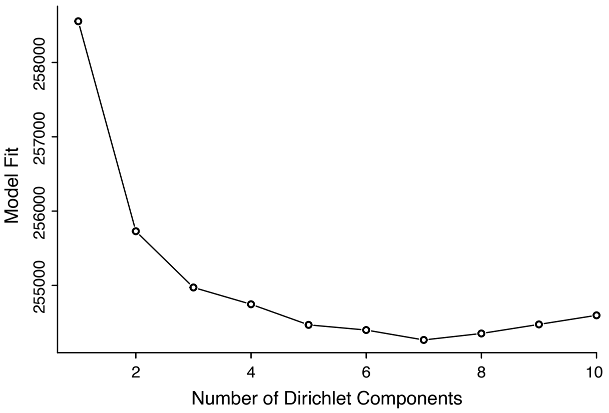

Supplementary Figure 2. Dirichlet Components. The optimal number of clusters (Dirichlet components) used to cluster the children samples to investigate temporal oral microbiota dynamics as the children age.

Supplementary Figure 3

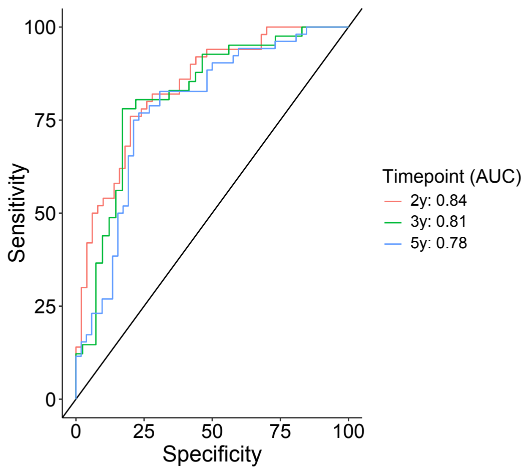

Supplementary Figure 3. sPLS-DA AUC. The area under the curve (AUC) from the sPLS-DA illustrating how well each bacterial score model separate children with and without caries at 5y. The analysis was performed on samples from children at 2y, 3y and 5y of age.
